# Improving the Dysregulation of FoxO1 Activity Is a Potential Therapy for Alleviating Diabetic Kidney Disease

**DOI:** 10.3389/fphar.2021.630617

**Published:** 2021-03-30

**Authors:** Yan Wang, Weichun He

**Affiliations:** Center for Kidney Disease, Second Affiliated Hospital, Nanjing Medical University, Nanjing, China

**Keywords:** forkhead box O1, diabetic kidney disease, posttranslational modification, sirtuin-1, oxidative stress

## Abstract

A substantial proportion of patients with diabetes will develop kidney disease. Diabetic kidney disease (DKD) is one of the most serious complications in diabetic patients and the leading cause of end-stage kidney disease worldwide. Although some mechanisms have been revealed to contribute to the understanding of the pathogenesis of DKD and some drugs currently in use have been shown to be beneficial, prevention and management of DKD remain tricky and challenging. FoxO1 transcriptional factor is a crucial regulator of cellular homeostasis and posttranslational modification is a major mechanism to alter FoxO1 activity. There is increasing evidence that FoxO1 is involved in the regulation of various cellular processes such as stress resistance, autophagy, cell cycle arrest, and apoptosis, thereby playing an important role in the pathogenesis of DKD. Improving the dysregulation of FoxO1 activity by natural compounds, synthetic drugs, or manipulation of gene expression may attenuate renal cell injury and kidney lesion in the cells cultured under a high-glucose environment and in diabetic animal models. The available data imply that FoxO1 may be a potential clinical target for the prevention and treatment of DKD.

## Introduction

**FIGURE 1:**
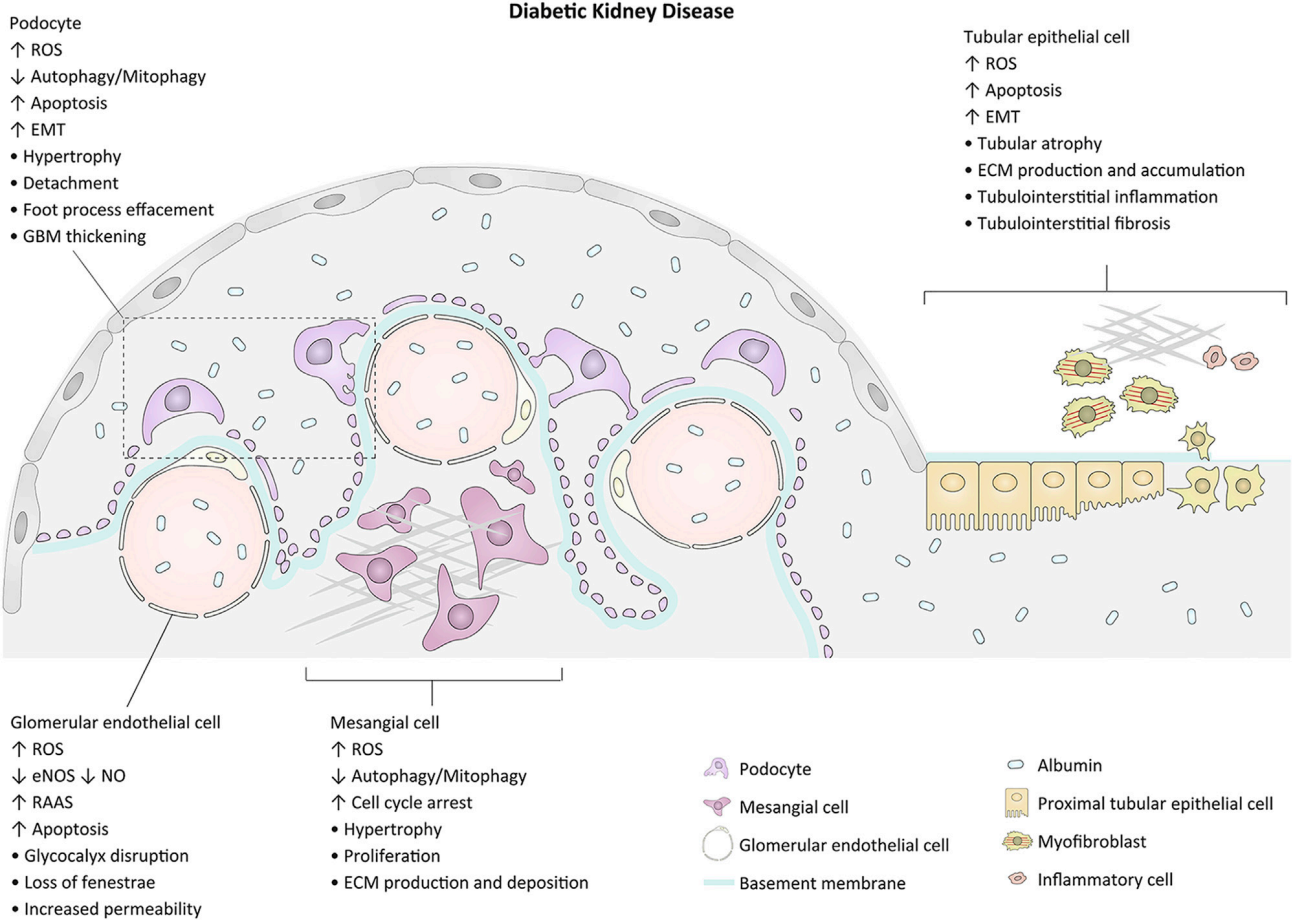
Major events and morphological changes related to the pathogenesis of glomerulopathy and tubulointerstitial lesion in diabetes. GBM, glomerular basement membrane; EMT, epithelial-mesenchymal cell transformation; ROS, reactive oxygen species; ECM, extracellular matrix.

Diabetic kidney disease (DKD), one of the common complications related to both types of diabetes, occurs in approximately 30–40% of diabetic patients and is the main cause of end-stage renal disease worldwide (Gnudi et al., 2016; Bonner et al., 2020; Chen et al., 2020). Renal enlargement and increased glomerular filtration rate are the initial changes of kidneys in diabetes. The earliest symptom is often albuminuria, which can develop into nephrotic-range proteinuria with morphological abnormalities such as glomerular hypertrophy, glomerular basement membrane (GBM) thickening, and extracellular matrix (ECM) expansion. Progressive glomerulosclerosis from nodular (Kimmelstiel-Wilson lesion) to global and tubulointerstitial fibrosis contributes to progressive loss of renal function in advanced DKD (Fioretto and Mauer, 2010; Tervaert et al., 2010; Badal and Danesh, 2014). The pathogenesis of DKD is multifactorial. The major pathophysiologic mechanisms contributing to glomerulopathy and tubulointerstitial lesions and the related morphological alterations are exhibited in Figure 1. Intensive management of patients with DKD including control of blood glucose and blood pressure, blockade of the renin-angiotensin-aldosterone system (RAAS), and inhibition of the sodium-glucose cotransporter 2 (SGLT2) may slow the progression of the disease. However, owing to the intricate pathogenesis of DKD, there is still no effective treatment to prevent the onset and to arrest the progression of the disease (Stanton, 2014; Thomas et al., 2015; Kidney Disease: Improving Global Outcomes Diabetes Work, 2020). Therefore, exploring the underlying mechanisms of renal impairment in the pathophysiological state of diabetes will be helpful to identify possible intervention targets and develop promising therapeutic strategies for DKD.

Forkhead box O (FoxO) transcription factors are essential modulators of cellular homeostasis. FoxO proteins respond to various external stimuli, including nutrient deprivation, growth factor signaling, oxidative stress (OS), and genotoxic stress. These input signals influence FoxOs intracellular localization, DNA binding, and interactions with other cofactors via a series of posttranslational modifications (PTM) containing phosphorylation, acetylation, ubiquitination, and methylation. Through integrating these modifications, FoxOs regulate cell-type-specific gene expression programs to respond to stress, maintain metabolic homeostasis, and balance redox (Tothova et al., 2007; Link and Fernandez-Marcos, 2017; Murtaza et al., 2017; Brown and Webb, 2018). There is growing evidence that, via the downstream target genes that are involved in the regulation of a variety of cellular processes such as energy metabolism, stress resistance, apoptosis, autophagy, and cell cycle arrest, FoxOs play a crucial role in the molecular mechanisms of DKD development, among which FoxO1 is the most extensively studied. This review summarizes our current perspectives on the regulation of FoxOs activity and the physiological functions of FoxO1, highlighting evidence to support the notion that dysregulated FoxO1 activity contributes toward renal parenchymal cell damage in the pathogenesis of DKD.

### The Regulation of FoxO Activity

There are four different FoxO transcription factors in mammals including FoxO1, FoxO3a, FoxO4, and FoxO6, which belong to the family of forkhead proteins. Each FoxO protein consists of four regions: a DNA-binding domain at N-terminal, a transactivation domain at C-terminal, a nuclear export sequence, and a nuclear localization sequence. All FoxO proteins share a common highly conserved DNA-binding domain, while other domains are enriched in sites for PTM and protein-protein interactions and are specific for the unique members (Tothova et al., 2007; Arden, 2008; Link and Fernandez-Marcos, 2017). In cells receiving survival or growth factor signal, the activity of FoxO protein is downregulated, primarily by being sequestered in the cytoplasm of the cell (Brunet et al., 1999; Brunet et al., 2002). The decrease in the level of FoxO protein may result from increased proteasomal degradation (Aoki et al., 2004). Upregulation of FoxO activity is by increased mRNA stability and expression as well as chromosomal rearrangement causing fusion of FoxO transactivation domain with DNA-binding domain of other transcription factors (Fritz and Radziwill, 2011).

The activity of FoxO protein is largely regulated by PTM that has been recognized as a crucial mechanism for the alteration of FoxO activity. Phosphorylation of FoxO by serine/threonine kinase Akt or serum- and glucocorticoid-induced kinase (SGK) exposes the nuclear export sequence and increases FoxO translocation to the cytoplasm, and the cytoplasmic sequestration or ubiquitination and subsequent proteasomal degradation inhibit FoxO activity (Brunet et al., 1999; Zhao et al., 2004; Huang et al., 2005; Huang and Tindall, 2011; Tzivion et al., 2011; Saline et al., 2019). Conversely, specific phosphorylation of FoxO by kinase mammalian sterile 20-like kinase 1 (MST1) or c-Jun N-terminal kinase (JNK) regulates FoxO activity in the opposite direction (Lehtinen et al., 2006; Densham et al., 2009; Brown and Webb, 2018). Reversible acetylation of FoxO regulates its activity as a second modulation layer. The acetylation of FoxO by cAMP-response element-binding protein (CREB)-binding protein (CBP), p300 or p300/CBP-associated factors (PCAF), and the subsequent deacetylation by class I and II histone deacetylases including the nicotinamide adenine dinucleotide (NAD^+^)-dependent deacetylase sirtuin-1 (Sirt1) alter the transcriptional activity of FoxO (Imai et al., 2000; Perrot and Rechler, 2005; Haigis and Sinclair, 2010). Acetylated FoxO is retained in the nucleus for engaging Sirt1, while deacetylation of FoxO by Sirt1 promotes FoxO transcriptional activity and accelerates FoxO degradation (Kitamura et al., 2005). Acetylation reduces the DNA-binding capacity of FoxO protein and enhances Akt-dependent phosphorylation of FoxO, suggesting the interplay between different PTM in regulating FoxO activity. Phosphorylation-dependent nuclear exclusion and deacetylation-dependent nuclear retention synergistically regulate the activity of FoxO protein, and each PTM may affect another (Matsuzaki et al., 2005; Qiang et al., 2010).

### The Physiological Function of FoxO1

FoxO binds via the DNA-binding domain to the same consensus binding site (5'-*TTGTTTAC*-3') within the promoter of its target gene. FoxO-DNA affinity differs between response element and PTM (Brent et al., 2008). The combination of FoxO with different sets of genes in different tissues results in a diversity of FoxO-mediated biological effects. The physiological functions of different FoxO proteins are not identical (Link and Fernandez-Marcos, 2017; Murtaza et al., 2017; Brown and Webb, 2018).

FoxO1, highly expressed in insulin-responsive tissues such as liver, adipose tissue, skeletal muscle, and pancreas, coordinates transcriptional cascades to modulate glucose metabolism and is therefore considered as a major governor of insulin signaling and glucose homeostasis. As a final effector of the insulin signaling pathway, FoxO1 responds in general to decreased nutrients by inducing gluconeogenesis in the liver, inhibiting adipocyte and myocyte differentiation, or shifting fuel utilization in muscle from glucose to lipids (Dong et al., 2008; Kousteni, 2012). In the absence of growth factor or insulin signaling or with stress stimuli, FoxO1 resides in the nucleus and is active as a transcription factor that governs apoptosis, autophagy, cell cycle arrest, stress resistance, and immune response. The program of gene expression transcriptionally regulated by FoxO1 ordinarily protects cells from the life-threatening consequences of nutrient, oxidative, or genotoxic stress (Dong et al., 2008; Murtaza et al., 2017).

### Dysregulation of FoxO1 Activity Is Involved in the Pathogenesis of Diabetic Kidney Disease

Several studies suggest that genetic variation in the FoxO1 gene is a predisposing factor for type 2 diabetes (T2D) or DKD in humans, revealing that FoxO1 may be involved in the initiation and development of DKD in patients with T2D, which provides new insight into the etiology of DKD (Müssig et al., 2009; Gong et al., 2017; Zhao et al., 2017).

Studies for investigating the protective effects of certain oral hypoglycemic drugs or natural compounds on the kidneys in diabetic animal models demonstrate that FoxO1 is an important target. Xu et al. reported that puerarin, a natural isoflavone from *Pueraria lobata* (Wild.), upregulated the expression of Sirt1, peroxisome proliferator-activated receptor *γ* coactivator 1α (PGC-1α), and FoxO1 in renal cortex from type 1 diabetic (T1D) mice. Puerarin reduced reactive oxygen species (ROS) and increased the activity of manganese superoxide dismutase (Mn-SOD) and catalase (CAT), accompanied by attenuated kidney tissue damage. These findings suggest that puerarin exerts renal protection effect on DKD through the Sirt1-PGC-1α/FoxO1 pathway (Xu et al., 2016). In a T2D rat model, liraglutide, a glucagon-like peptide-1 agonist, markedly reduced renal damage including the production of ECM proteins. Liraglutide inhibited the phosphorylation of FoxO1 and increased Mn-SOD expression in the diabetic kidneys. It seems that liraglutide exerts a protective effect on early DKD by a FoxO1-mediated upregulation of renal Mn-SOD (Chen et al., 2018). Hussein et al. reported that treatment of DKD rats with the Sirt1 agonist resveratrol increased superoxide dismutase (SOD) activity and reduced malondialdehyde (MDA), collagen (Col) IV, and fibronectin (FN) expression by increasing FoxO1 activity (Hussein and Mahfouz, 2016).

In addition to these *in vivo* studies, many studies have investigated the effect of the change in FoxO1 activity on renal parenchymal cell injury in diabetic conditions by *in vitro* and *in vivo* experiments, as shown below by the different cell types studied.

#### Podocyte

Podocyte is one of the components of the glomerular filtration barrier (GFB) and plays an essential role in maintaining the integrity of GFB. As a terminal differentiated atypical epithelial cell, podocyte cannot regenerate after suffering from injury and apoptosis (Quaggin and Kreidberg, 2008). Under a diabetic environment, the podocyte often undergoes hypertrophy, epithelial-mesenchymal cell transformation (EMT), apoptosis, and detachment, leading to the impairment and destruction of GFB, which becomes a crucial constituent in the development of DKD (Figure 1) (Fioretto and Mauer, 2010; Tervaert et al., 2010; Oh et al., 2012; Dai et al., 2017).

The glomerular insulin signaling is critical for GFB integrity and normal kidney function (Welsh et al., 2010), and podocyte is a unique insulin-responsive cell in the GFB (Coward et al., 2005). The insulin-dependent phosphorylation of Akt was impaired in the podocytes from diabetic mice at the onset of albuminuria. Dysregulation of Akt phosphorylation and subsequent FoxO1 phosphorylation in podocytes was associated with its susceptibility to apoptosis, suggesting that the inability of podocyte to respond to insulin partially accounts for the decreased podocyte number seen in early DKD (Tejada et al., 2008; Katsoulieris et al., 2016).

Several studies investigated the effect of FoxO1 on protecting podocytes from injury in diabetes. The transcriptional activity of FoxO1 decreased in the kidney from type 1 diabetic rodents induced by streptozotocin (STZ) and the podocytes cultured under high-glucose (HG) condition (Guo et al., 2015; Du et al., 2016; Li et al., 2016; Li et al., 2017). Guo et al. found that overexpressing FoxO1 by injection of recombinant lentivirus into the renal cortex decreased albuminuria and serum urea nitrogen and creatinine levels, preserved podocalyxin and nephrin expression, and ameliorated pathological changes in the glomerulus of diabetic kidneys, suggesting the protective effect of FoxO1 on podocyte injury (Guo et al., 2015). Li et al. reported that upregulation of FoxO1 activity reversed HG-dependent downregulation of PTEN-induced putative kinase 1 (PINK1), an important functional protein in mitophagy, which suggests that, through downstream PINK1/Parkin pathway, FoxO1 limits the production of ROS under HG conditions and maintains mitochondrial morphology and stability, thus playing a crucial role in the protection against mitochondrial dysfunction and podocyte apoptosis (Li et al., 2016; Li et al., 2017). Du et al. found that constitutive FoxO1 activation suppressed HG-induced activation of the transforming growth factor (TGF)-β1/Smad3/integrin-linked kinase (ILK) pathway and thus partially reversed podocyte EMT (Du et al., 2016).

A number of studies have revealed that the renoprotective effect of certain drugs used to treat diabetes or some protein molecules is FoxO1-mediated. For example, FoxO1 was identified as a target of microRNA (miR)-21, and the upregulation of miR-21 in podocytes cultured under HG conditions inhibited the expression of FoxO1, thus attenuating autophagy and promoting apoptosis (Wang et al., 2019a). While Atrasentan, an endothelin-1 receptor antagonist (Egido et al., 2017), could enhance FoxO1 expression by downregulating miR-21 and thereby attenuate HG-induced podocyte injury and hamper the progression of DKD (Wang et al., 2019a). In another study, progranulin (PGRN), a secreted glycoprotein, attenuated mitochondrial damage and dysfunction in the podocytes treated with HG. Since PGRN induced the expression of Sirt1 and reduced the acetylation levels of PGC-1α and FoxO1 in HG-treated podocytes, it suggests that PGRN modulates mitochondrial biogenesis and mitophagy through Sirt1-PGC-1α/FoxO1 signaling and thus protects against podocyte injury in DKD (Zhou et al., 2019).

#### Mesangial Cell

Mesangial cell (MC) plays an important role in maintaining the structural integrity of glomerular capillary and mesangial matrix homeostasis. MC also can regulate filtration surface area and phagocytose apoptotic cells or immune-complexes. MC hypertrophy and mesangial matrix expansion are among the earliest pathological features of DKD. MCs are primary targets of diabetes and they respond differently to a diabetic environment, where some of them acquire an activated phenotype undergoing hypertrophy and proliferation with excessive production of matrix proteins, growth factors, chemokines, and cytokines, whereas others undergo apoptosis (Figure 1) (Abboud, 2012).

Das et al. found that HG induced Akt-dependent phosphorylation of FoxO1, and dominant-negative FoxO1 increased the phosphorylation of Akt. CAT blocks HG-stimulated Akt phosphorylation to inhibit the inactivation of FoxO1 and PRAS40, leading to the inhibition of mTORC1 activity. In contrast, HG-inactivated FoxO1 decreased CAT expression, leading to an increase in ROS production, mTORC1 activation, MC hypertrophy, and FN and PAI-1 expression. These findings suggest the existence of a positive feedback loop involving sustained Akt activation, FoxO1 inactivation, decreased CAT expression, and increased ROS, resulting in mTORC1 activation, MC hypertrophy, and matrix excessive production (Das et al., 2014).

Wu et al. reported that HG-induced FoxO1 inhibition and relevant PGC-1α downregulation were accompanied by mitochondrial dysfunction and increased ROS generation, whereas constitutive FoxO1 activation increased PGC-1α expression and partially reversed these changes in MCs. PGC-1α was identified as a direct transcriptional target of FoxO1. Overexpression of FoxO1 in diabetic rat kidneys significantly increased the expression of PGC-1α, mitochondrial-related transcription factor (Nrf1), and mitochondrial fusion protein (Mfn2) and decreased MDA production and proteinuria. These findings suggest that the activation of FoxO1/PGC-1α attenuated HG-induced mitochondrial dysfunction and MC injury (Wu et al., 2015).

Guo et al. found that HG-elevated p-Akt level and subsequent alleviation of FoxO1 activity were accompanied by the downregulation of CAT and SOD2 mRNA expression, activation of TGF-β/Smad signaling, and increases in the protein expression of FN and Col I in MCs. Conversely, overexpression of nucleus-localized FoxO1 upregulated the expression of antioxidative enzymes, accompanied by inhibition of TGF-β/Smad3 signaling and a decrease in the expression of ECM proteins. This study suggests that the antioxidative effect mediated by FoxO1 may play a crucial role in attenuating TGF-β-induced ECM production in MCs under an HG environment (Guo et al., 2016).

Fiorentino et al. found that tissue inhibitors of metalloproteinase3 (TIMP3), an inhibitor of ADAM metallopeptidase domain 17 (ADAM17), were reduced in the kidneys from type 1 diabetic mice. In the kidneys of diabetic *Timp3*-deficient mice, the expression of FoxO1 and FoxO1-targeted autophagy-related genes, including Atg5, Atg8, LC3, and Beclin1, was decreased, and the expression of signal transducers and activator of transcription 1 (STAT1), a repressor of FoxO1 transcription, was increased. Renal biopsy of patients with DKD showed similar data. Knockdown of TIMP3 in the MCs cultured under an HG environment led to the downregulation of FoxO1 and FoxO1-targeted autophagy-related genes and an increase in the LC3II/I ratio. This study suggests that the reduction of autophagy, especially in MCs, caused by TIMP3 deficiency may deteriorate DKD (Fiorentino et al., 2013).

Liu et al. reported that overexpression of FoxO1 in MCs caused upregulation of p27 and downregulation of cyclin D1 and CDK4, which promoted cell cycle arrest at the G0/G1 phase and attenuated proliferation induced by HG. Degradation of FoxO1 caused a decrease in p27 and stimulated MCs proliferation. These findings suggest that FoxO1 is involved in regulating MCs proliferation induced by HG via FoxO1/p27 signaling (Liu et al., 2014).

In a recent study, metformin effectively attenuated glycolipid metabolic disorders as well as renal damage in a T2D rat model. Mechanistically, metformin relieved OS, enhanced autophagy, and suppressed cell proliferation in cultured MCs stimulated by HG through AMPK/Sirt1-FoxO1 signaling pathway (Ren et al., 2020).

#### Glomerular Endothelial Cell

The glomerular endothelial cell (GEC), which is highly fenestrated and covered by a rich glycocalyx, participates in the sieving properties of GFB and in the maintenance of podocyte structure. Both a reduction in the thickness of the glycocalyx and a reduction in the fenestration of endothelium are early characteristics of DKD. GEC injury can occur via hemodynamic stimuli that cause reduced nitric oxide (NO) bioavailability via suppression of endothelial nitric oxide synthase (eNOS), or it can result from growth factor driven altered metabolism. As GEC is the first cell encountered by any circulating stimulus relevant to diabetes, it not only is a direct target of diabetes but also serves as cell sending paracrine signals to adjacent MC and podocyte (Figure 1) (Dane et al., 2013; Jourde-Chiche et al., 2019).

Carota et al. reported that the expression of vascular endothelial protein tyrosine phosphatase (VE-PTP), which can dephosphorylate tyrosine kinase with Ig and EGF homology domains 2 (TIE2), was robustly upregulated in the GECs in a diabetic mouse model. The reduction of TIE2 signaling due to increased VE-PTP expression under diabetic conditions resulted in decreased eNOS phosphorylation, as well as increased FoxO1 levels and its downstream profibrotic and proinflammatory targets (Carota et al., 2019). In this study, FoxO1 appears to play an opposite role in GECs than in other renal parenchymal cells and reduced transcriptional activity of FoxO1 and subsequent downstream target genes expression may ameliorate GECs injury in diabetic rodents. The mechanism of FoxO1 activity regulation in GEC and the effect of FoxO1 on GEC injury under diabetic conditions need further studies.

#### Proximal Tubular Epithelial Cell

Although glomerulosclerosis is a major feature of DKD, the severity of tubulointerstitial lesions ultimately determines the extent of renal impairment. Albuminuria, a hallmark of DKD, can activate proximal tubular epithelial cell (PTEC) to evoke tubulointerstitial inflammation (Tang et al., 2003). In addition to albumin, several diabetes-related substrates such as HG, advanced glycation end-products, and angiotensin II may activate a number of signaling pathways including nuclear factor kappa B, extracellular signal-regulated kinase 1/2, p38 mitogen-activated protein kinases, protein kinase C, STAT1, and ROS generation, leading to the accumulation of numerous growth factors, cytokines, chemokines, and adhesion molecules in the interstitium to orchestrate further inflammation and fibrosis (Figure 1) (Donadelli et al., 2003; Tang et al., 2003; Tang and Lai, 2012).

Thioredoxin-interacting protein (TXNIP) is a negative regulator of thioredoxin (TRX). TXNIP-TRX has been shown to be an important contributor to the enzyme system involved in ROS production and renal OS (Li et al., 2009; Kibbe et al., 2013). Ji et al. reported that TXNIP and TXN were identified as the direct FoxO1 transcriptional targets, and kidney-specific overexpression of FoxO1 attenuated renal tubular injury by restraining the increase in TXNIP and the decrease in TRX levels in diabetic mice. The study suggests that FoxO1 protects against HG-induced renal PTECs injury through regulating TXNIP-TRX-mediated ROS generation (Ji et al., 2019).

The activation of STAT1, the phosphorylated form of STAT1 (p-STAT1), has been shown to be involved in tubular EMT and tubulointerstitial fibrosis (TIF) in animal models including diabetes (Nakajima et al., 2004; Nightingale et al., 2004). Huang et al. reported that kidney-specific overexpression of FoxO1 significantly downregulated p-STAT1, accompanied by reduced renal damage, apoptosis, and TIF in diabetic mice. Knockdown of FoxO1 in PTECs enhanced the expression of p-STAT1, resulting in EMT and apoptosis, whereas overexpression of FoxO1 markedly inhibited EMT and apoptosis in PTECs under an HG environment. These findings suggest that, partially through STAT1 signaling, FoxO1 plays a protective role against PTECs injury in DKD (Huang et al., 2019).

The effects of Sirt1 on suppressing apoptosis induced by kidney cell injuries, alleviating renal inflammation, improving mitochondrial function, and repressing OS indicate that it is involved in the development of DKD (Dong et al., 2014; Wang et al., 2019b). Zhou et al. reported that HG induced PTECs injury by attenuating the deacetylase activity of Sirt1 (Zhou et al., 2015). Further study showed that metastasis-associated lung adenocarcinoma transcript 1 (MALAT1), which belongs to the long noncoding RNA (lncRNA), was upregulated in the kidney from diabetic mice and in the PTECs cultured with HG, and the expression of Sirt1 was decreased. The interaction between MALAT1 and FoxO1 was promoted by HG. By combination with the promoter of Sirt1, FoxO1 induced Sirt1 transcription, whereas MALAT1 repressed Sirt1 expression by targeting FoxO1. These findings suggest that the interaction between lncRNA MALAT1 and FoxO1 represses the transcription of Sirt1 in PTECs treated with HG and thus promotes HG-induced PTECs injury (Zhou et al., 2018).

## Discussion

**FIGURE 2:**
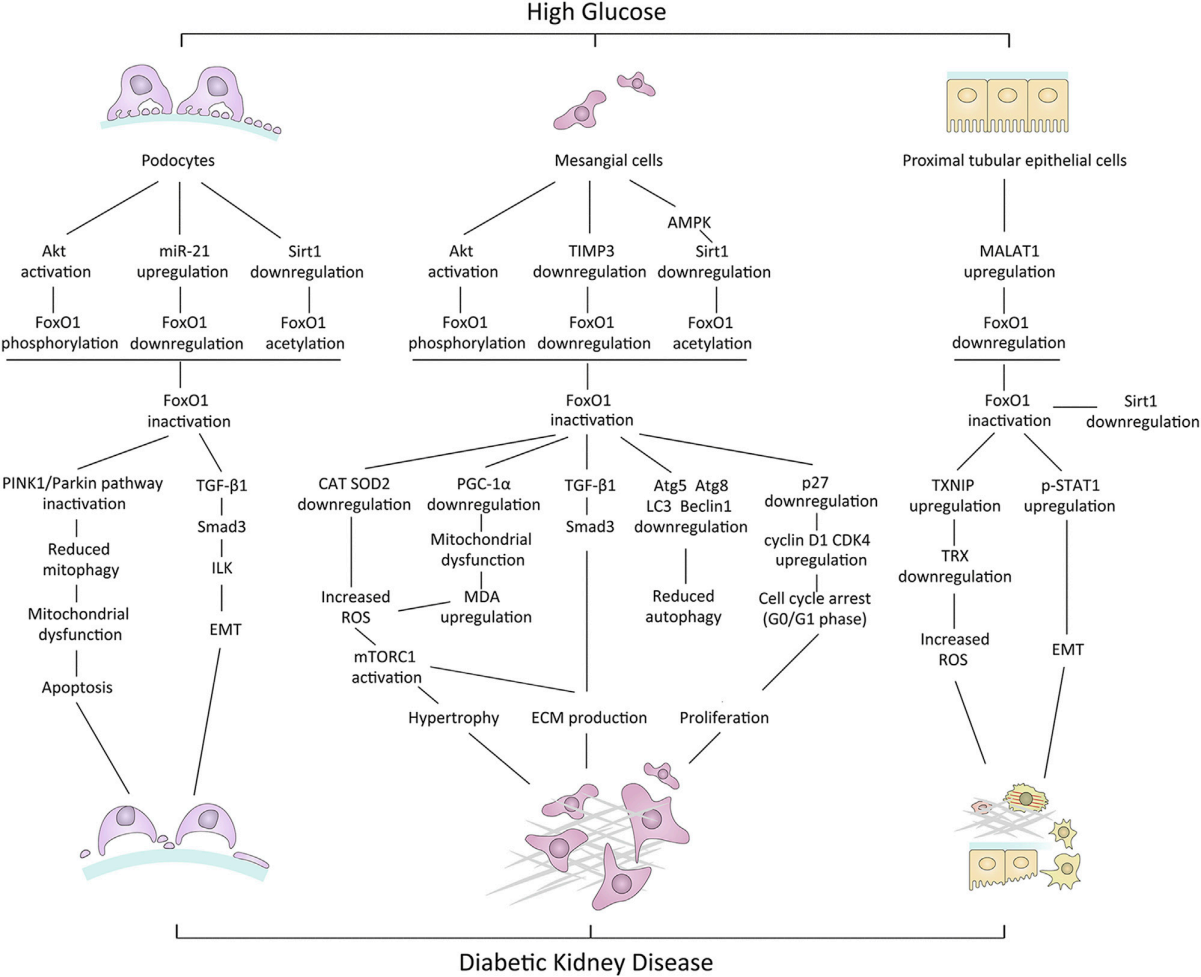
Mechanisms underlying renal cell damage associated with dysregulation of FoxO1 activity in high-glucose conditions. PINK1, PTEN-induced putative kinase 1; TGF-β1, transforming growth factor-beta1; ILK, integrin-linked kinase; EMT, epithelial-mesenchymal cell transformation; TIMP3, tissue inhibitors of metalloproteinase3; CAT, catalase; SOD, superoxide dismutase; MDA, malondialdehyde; ROS, reactive oxygen species; ECM, extracellular matrix; TXNIP, thioredoxin-interacting protein; TRX, thioredoxin; p-STAT1, phosphorylated STAT1.

Metabolic disturbance, mitochondrial dysfunction, OS, inflammation, impaired autophagy, and apoptosis may contribute to diabetic renal cell injury. Data from animal models and cell experiments suggest that the dysregulation of FoxO1 activity may be associated with these cellular processes, leading to kidney damage in the diabetic environment, thereby being involved in the pathogenesis of DKD. Mechanisms underlying renal cell damage associated with dysregulation of FoxO1 activity in HG conditions are summarized in Figure 2.

**FIGURE 3:**
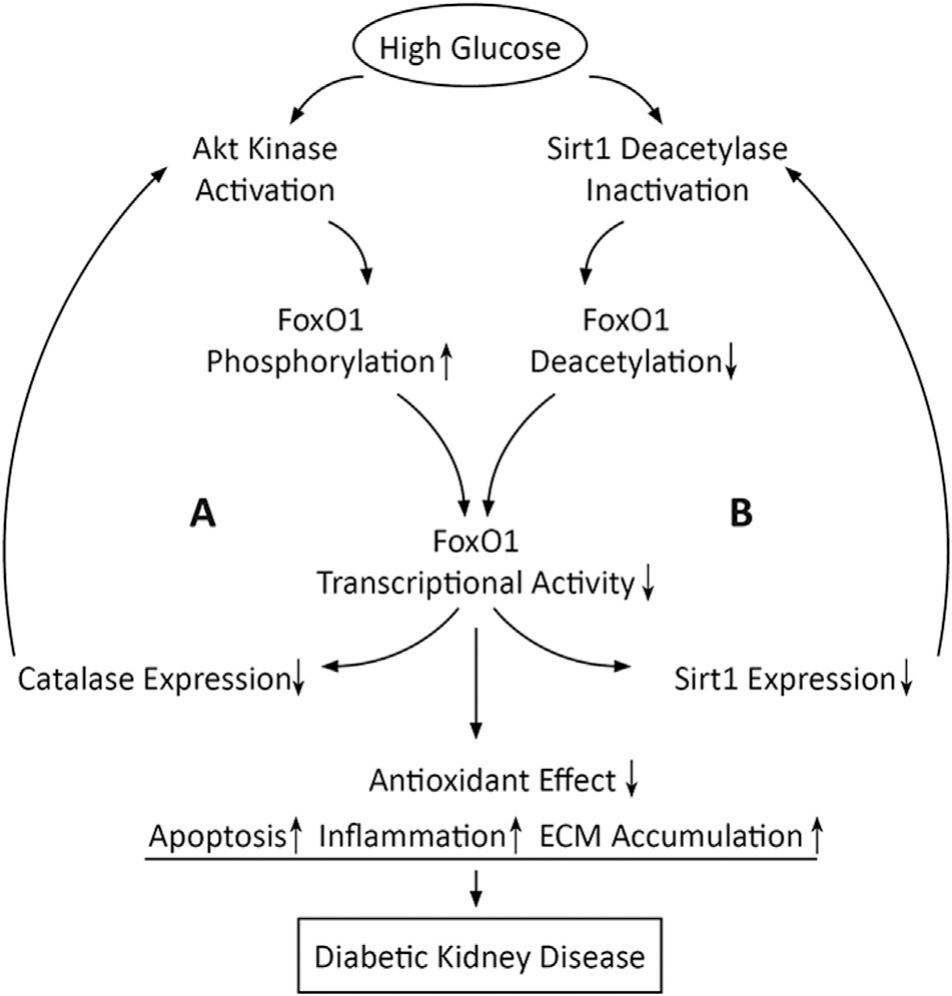
Schematic diagram shows that high glucose may induce two positive feedback loops in which FoxO1 inactivation is involved. Loop A is positive feedback between activation of Akt and inactivation of FoxO1 and loop B is positive feedback between inactivation of Sirt1 and inactivation of FoxO1.

PTM is an important mechanism for regulating FoxO activity, and the abnormality in PTM in diabetic conditions is the common reason for FoxO1 dysfunction. Kidney is an important target organ of insulin action (Lay and Coward, 2018). The disruption of normal insulin signaling owing to hyperinsulinemia, insulin resistance, or absolute insulin deficiency associated with diabetes causes dysregulation of FoxO1 phosphorylation and subcellular localization, leading to improper FoxO1 activity and its target genes transcription (Katsoulieris et al., 2016). Furthermore, the inactivation of FoxO1 owing to Akt-dependent phosphorylation and the phosphorylation of Akt owing to FoxO1 inactivation are mutually reinforcing, which results in a positive feedback between activation of Akt and inactivation of FoxO1 in diabetic conditions (Das et al., 2014). On the other hand, the reduced deacetylase activity of Sirt1 by HG inhibits transcriptional activity of FoxO1 (Wang et al., 2019b), while the decreased transcriptional activity of FoxO1 causes the reduced Sirt1 transcription and expression, which seems also to be positive feedback in diabetic conditions (Zhou et al., 2018) (Figure 3).

Based on the majority of the available evidence, dysregulation of FoxO1 activity may reduce the antioxidant effect to respond to OS, leading to apoptosis, inflammation, and ECM accumulation in diabetic kidneys. Given that FoxO1 inactivation induced by high glucose may be enhanced unceasingly even if hyperglycemia is controlled, it is conceivable that the self-reinforcement of the FoxO1 activity dysregulation could be one of the reasons for the progression of kidney damage in patients with DKD who have well levels of blood glucose. Therefore, natural compounds or synthetic drugs that can modulate the activity of FoxO1 could be a novel therapeutic option for alleviating DKD. Potential FoxO1 modulators, their cellular targets, and their effects on cell physiology are summarized in Table 1.

**TABLE 1:** Potential FoxO1 modulators and their effects in DKD.

Modulators	Cellular targets	Effects on FoxO1-mediated pathway and cell physiology	Experimental model of DKD	References
Puerarin	↑Sirt1 expression	↑PGC-1α/FoxO1 deacetylation ↓ROS ↑Mn-SOD and CAT activity	T1D mice	Xu et al. (2016)
Liraglutide	↓FoxO1 phosphorylation	↑FoxO1 activity ↑Mn - SOD expression ↓ECM production	T2D rats	Chen et al. (2018)
Resveratrol	↑Sirt1 activity	↑FoxO1 deacetylation ↑SOD activity ↓MDA expression ↓Col IV and FN expression	T2D rats	Hussein and Mahfouz (2016)
Atrasentan	↓miR-21 expression	↑FoxO1 expression	Podocytes cultured in HG, T2D (KK-Ay) mice	Wang et al. (2019a)
Progranulin	↑Sirt1 expression	↑PGC-1α/FoxO1 deacetylation ↓Mitophagy ↑Mitochondrial biogenesis	Podocytes cultured in HG, T1D mice	Zhou et al. (2019)
Metformin	↑AMPK/Sirt1	↑FoxO1 activity ↓ROS ↑Autophagy ↓Cell proliferation	Mesangial cells cultured in HG, T2D rats	Ren et al. (2020)

T1D, type 1 diabetes; T2D, type 2 diabetes; Mn-SOD, manganese superoxide dismutase; CAT, catalase; SOD, superoxide dismutase; MDA, malondialdehyde; ROS, reactive oxygen species; Col, collagen; FN, fibronectin.
